# A Review of Control Strategies in Closed-Loop Neuroprosthetic Systems

**DOI:** 10.3389/fnins.2016.00312

**Published:** 2016-07-12

**Authors:** James Wright, Vaughan G. Macefield, André van Schaik, Jonathan C. Tapson

**Affiliations:** ^1^Biomedical Engineering and Neuroscience, The MARCS Institute, University of Western SydneySydney, NSW, Australia; ^2^School of Medicine, University of Western SydneySydney, NSW, Australia; ^3^Neuroscience Research AustraliaSydney, NSW, Australia

**Keywords:** neuroprosthetics, control theory, closed-loop, brain-machine interface, feedback

## Abstract

It has been widely recognized that closed-loop neuroprosthetic systems achieve more favorable outcomes for users then equivalent open-loop devices. Improved performance of tasks, better usability, and greater embodiment have all been reported in systems utilizing some form of feedback. However, the interdisciplinary work on neuroprosthetic systems can lead to miscommunication due to similarities in well-established nomenclature in different fields. Here we present a review of control strategies in existing experimental, investigational and clinical neuroprosthetic systems in order to establish a baseline and promote a common understanding of different feedback modes and closed-loop controllers. The first section provides a brief discussion of feedback control and control theory. The second section reviews the control strategies of recent Brain Machine Interfaces, neuromodulatory implants, neuroprosthetic systems, and assistive neurorobotic devices. The final section examines the different approaches to feedback in current neuroprosthetic and neurorobotic systems.

## Introduction

A neuroprosthetic is a device or system that has an interface with the nervous system and supplements or substitutes functionality in the patient's body. For the purpose of this review we have included neuromodulatory systems and brain computer interfaces under the general description of neuroprosthetics, as well as rehabilitation systems such as exoskeletons. The key identifying characteristic of the neuroprosthetic is that it has an interface with the subject's nervous system, as distinct from an implantable devices such as an pacemaker or an insulin pump. Consequently there are a broad range of devices that we consider neuroprosthetics. To date there have been a number of reviews of neuroprosthetic systems. There is significant literature on classification algorithms and detection strategies (Schwartz, 2004; Lotte et al., 2007; Green and Kalaska, 2011; Borton et al., 2014; Morimoto and Kawato, 2015), including comparison and evaluation of the relative strengths of different approaches. However, there are fewer examinations the authors are aware of that investigate the different control approaches that have been implemented in neuroprosthetic settings. Performing such a review is made more difficult due to the small number of studies that have compared different control approaches within the same experiment, and the fact that many neuroprosthetic studies have by necessity been conducted with very small sample sizes, sometimes involving a single subject. Additionally many of the devices examined in this review are experimental or investigational, and are not yet in use in clinical or therapeutic settings (Sun and Morrell, 2014).

**Figure 1:**
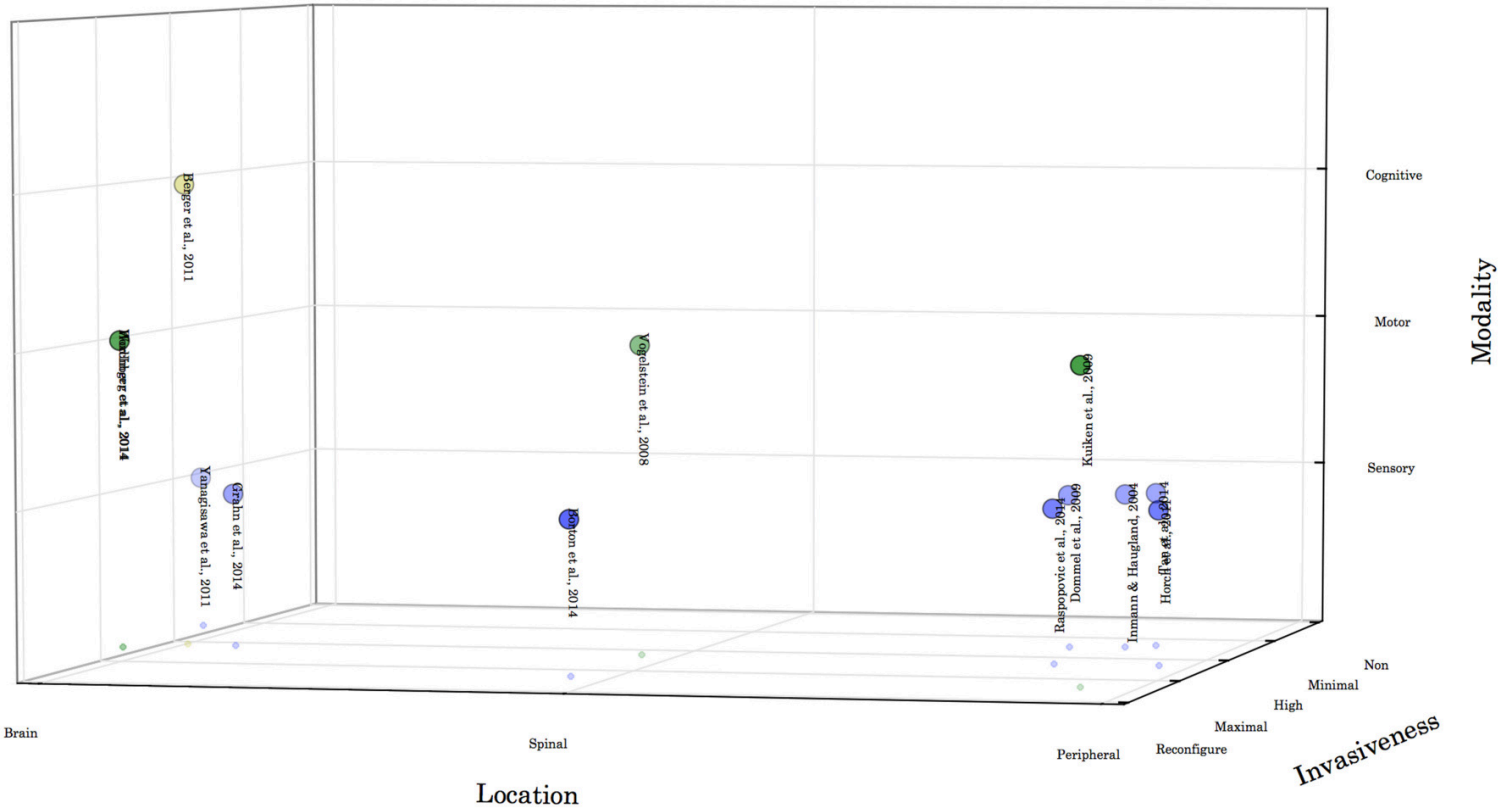
**Neuroprosthetic Systems**. An illustration of the conceptual space of neuroprosthetic devices. Devices can be classified as similar if they provide assistance in the same Modality, have an equivalent level of Invasiveness, or interface with the user's nervous system in the same Location.

When considering these devices in the context of control and feedback it can be helpful to place them along a number of axes (Figure 1) to partition the large variety of systems and approaches. The first axis we have considered is the location of the interface with the nervous system, with the Central Nervous System (CNS) subdivided into the brain and the spinal cord, and then the Peripheral Nervous System (PNS), consisting of afferent and efferent pathways. Along this axis and additional distinction can be drawn between single channel systems that use a single electrode as the interface to the subject's nervous system, and multichannel systems which utilize many parallel channels for interface. Finally the channel can be unidirectional for simplex communication, or bidirectional for half-duplex, or full duplex communication.

Another axis to consider is the invasiveness of the interface. At one end we place noninvasive interfaces such as Electroencephlogram (EEG), Magnetic Resonance Imaging (MRI), and Electromyography (EMG). These interfaces are hampered by a variety of issues, including low spatial resolution and low signal to noise ratios. The advantage they enjoy is that they can be deployed with little risk, which has meant they are a popular platform for neuroprosthetic development. Next are minimally invasive interfaces such as microwire EMG and microneurographic recordings from the PNS. With a more invasive interface there are better quality signals, but still minimal intervention with the subject's body, reducing risk of complications. These interfaces are susceptible to movement, leading to changes in the quality of the recorded signal. The most invasive interfaces require surgical implantation, and come with risks associated with surgery as well as risk of damage to the part of the nervous system. Within this category a distinction can be drawn between the less invasive, such as cuff electrodes around PNS sites and Electrocorticogram (ECog)—these interfaces do not disrupt the blood brain barrier; and the more invasive Multi Electrode Arrays (MEA) in PNS nerves, motor or somatosensory cortex, Deep Brain Stimulators (DBS) to treat Parkinson's Disease with electrodes in the Globus Pallidus, Sub Thalamic Nucleus, or the Pedunculopontine Nucleus. These multichannel interfaces offer high resolution, but comparatively small spatial coverage (Krook-Magnuson et al., 2015). These electrodes come with risks that are still not fully understood. These primarily involve the physical trauma due to the insertion of the electrode, with effects occurring over different timescales. Shortly after insertion there is bleeding and swelling, as well as physical damage to neurons (Fernandez et al., 2014). Over longer timescales the presence of the recording device compromises the blood-brain barrier, allowing ingress of cellular and molecular components from elsewhere in the body (Schwartz, 2004). Additionally the long term stability of the recording site is often compromised, due to immune and mechanical actions on the microelectrodes (Krook-Magnuson et al., 2015). At the furthest extent of this axis are the interfaces that reconfigure the subject's nervous system. Targeted Muscle Reinnervation (TMR) surgically rewires an amputee subject's PNS, by deinnervating muscles that have no biomechanical role after the amputation and redirecting the preserved nerves from the amputated limb to the deinnervated muscles (Kuiken et al., 2009), allowing for high quality EMG recordings as control inputs to a prosthetic. Optogenetic techniques offer a non-electrical interface to neurons, by using light to activate special ion-channels. This technique enables individual neurons to be targeted, which is extremely difficult with cortical microelectrodes, as well as the possibility of selectively activating a class of neurons such as excitatory neurons instead of inhibitory (Krook-Magnuson et al., 2015). However, optogentic approaches require the introduction of genes that encode for light activated ion-channels (Deisseroth, 2011), and the issues associated with this may preclude this technique in humans.

The final axis is the modality of the prosthesis. Sensory neuroprosthetics offer input from artificial sensors, as in a cochlear implant or bionic eye, or modulate sensory input as is the case in neurostimulators for treating chronic pain. Systems to treat motor impairment are frequently referred to as Brain Machine Interfaces (BMI) or Brain Computer Interfaces (BCI) and infer motor intent from the subject in order to control a virtual or physical effector. Cognitive devices modulate the activity of the CNS and include devices such as DBS stimulators for Parkinson's Disease, depression, and hippocampal stimulators for memory.

## Neuroprosthetics

The following examples are representative, but not exhaustive, and are placed in Figure 1 to illustrate the wide variety of devices that can be described as having a neuroprosthetic interface. Many of these devices are experimental demonstrations, and not clinically approved interventions. Adjacent to the choice of controller, there are choices about the location and method for acquiring a suitable input signal (Grill et al., 2009; Andersen et al., 2010), and questions about the consequences of long term use of these and similar devices that have not yet been answered.

Peripheral nervous system interfaces are attractive as they are less invasive than the central nervous system alternatives, while still offering a rich source of information for neuroprosthetic control. Inmann and Haugland (2004) used a cuff electrode around the median nerve to record nerve activation due to touch and that input was used to modulate the Functional Electrical Stimulation of the subjects muscles. Horch et al. (2011) demonstrated that human subjects who were provided with intrafascicular electrical stimulation of the median and ulnar nerves derived from sensors on a myoelectric prosthetic limb were able to use the feedback to perform object recognition tasks by digital manipulation. Tan et al. (2014) used non-penetrating cuff electrodes on the median, ulnar and radial nerves of human subjects to provide natural sensations of touch while operating a myoelectric prosthetic allowing for improved performance of manual tasks. Raspopovic et al. (2014) showed that a bidirectional interface with the median and ulnar nerves could be used to provide artificial sensory feedback related to the forces exerted on a sensorized prosthetic limb, and that the artificial sensations allowed the subject to improve their ability to sense characteristics of the objects being manipulated. Targeted Muscle Reinnervation generates a rich high density signal for surface Electromyography (EMG) that enables simultaneous operation of multiple degrees of freedom in a myoelectric prosthetic limb (Kuiken et al., 2009). The tissue serves as a bioamplifier for the nerve signal, allowing a large array of surface electrodes to be deployed on the subject. The array provides a rich signal suitable for pattern recognition, and combined with a high performance prosthetic limb gives the subject an improved experience. As a sensory modality prosthetic, (Dommel et al., 2009) are testing a vision prosthesis for electrical stimulation of the retina. Spinal cord stimulation may be able to generate gait patterns suitable for locomotion in paralyzed patients. Vogelstein et al. (2008) describes the design of a system that is capable of generating primitive locomotion in anesthetized felines. Borton et al. (2014) developed an electrochemical spinal neuroprosthesis to reactivate the circuits in a damaged spinal cord, allowing hindlimb movement sufficient to enable walking in paralyzed rats.

Yanagisawa et al. (2011) used ECog electrodes placed over the sensorimotor cortex of a stroke patient in order to control a supernumerary robotic hand that was able to mimic the hand posture of the subject. Berger et al. (2011) implanted microwire electrodes in the hippocampus of rats and recorded the activity while the animals were trained to complete a memory task. Subsequent stimulation of the electrodes according to the trained model improved the performance of the rats at the cognitive task. Neuromodulators for Deep Brain Stimulation have been used to treat the symptoms of Parkinson's disease and depression (Grahn et al., 2014). Multi Electrode Arrays implanted in motor cortex have been successfully used to acquire signals for the multi degree of freedom control of robotic limbs (Hochberg et al., 2012; Wodlinger et al., 2015). Guggenmos et al. (2013) describe a Brain Machine Brain Interface in rats that utilizes microwire recordings from a premotor area to detect spiking activity leading to stimulation of the somatosensory area. The Activity Dependant Stimulation via the neuroprosthetic prototype enabled rats with injury to the motor area to recover reach and grasp behavior.

This list of devices is not exhaustive, and serves only to illustrate the different axes of Modality, Invasiveness and Location when considering neuroprosthetic designs. For a more thorough description of the available neuroprosthetic devices and interface technologies, the reader is directed to the following excellent reviews (Navarro et al., 2005; Grill et al., 2009; Micera et al., 2010; Ortiz-Catalan et al., 2012). These reviews focus on the interface techniques with the nervous system, and provide a detailed discussion of the limits of current interfaces.

## Introduction to control

The design of a neuroprosthetic varies significantly between different modalities. Because it is a multidisciplinary field often the language used to describe the system can vary between devices. The terms “closed-loop,” “feedback,” and “online” may take on different meanings. Identifying and acquiring a suitable input signal is a non-trivial task (Krook-Magnuson et al., 2015). This makes the fabrication of a substitute system in the case of impairment a complex endeavor. Developing a suitable simplified model for embodiment as an open-loop system is often a first step. But it is not always straightforward; take motor control as example—many models of muscles exist. Highly biophysical models (such as cross-bridge models) of muscles become large systems of non-linear differential equations when describing whole muscles or limbs (Ionescu and De Keyser, 2006). Similarly modeling the individual neurons that act as part of the Basal Ganglia, in a complex network of interconnections across the CNS (Broccard et al., 2014), rapidly becomes an intractable problem when developing an open-loop model for DBS to treat Parkinson's Disease. As a consequence the Basal Ganglia has often been modeled internally by a neurologist (Hosain et al., 2014), rather than explicitly within the DBS device, with stimulation parameters adjusted by the clinician observing the patient's symptoms instead of in response to a model. It is also possible that the control problem for a given neuroprosthetic has more than a single loop that needs to be considered, possibly due to the interaction of different physiological systems and different timescales (Houk, 1988). In this case control may best be achieved by a hierarchy of controllers, or a series of adaptive controllers that can be tuned at different stages of design (McFarland et al., 2006).

### Nervous system control

All parts of the human body have evolved to operate by extremely complex closed-loop control. Different subsystems, such as the cardiovascular system or the immune system operate under closed-loop control, with sensors and effectors operating at micro (LeDuc et al., 2011) and macroscopic scales (Houk, 1988). Sensory organs can be directly connected to the nervous system, such as stretch receptors, or they can be indirectly coupled by messenger systems such as hormone signaling. Command of these systems can be voluntary, or have a voluntary component, but they may also be completely automatic. Substituting or supplementing the performance of an element of the body is the aim of a neuroprosthetic device. Achieving this involves the fabrication of an effector, such as a prosthetic limb, that can replicate at least a subset of the body's functionality. But there is no function without control, so it is also necessary to model, and potentially integrate with, the different control loops within the human body. There has been some good success with simpler systems, possibly due to the high level state abstraction of the control within the nervous system (Holinski et al., 2013), as in the control of the hand. By examining the joint angles for fingers during different hand postures (grasping different objects) using principal component analysis it has been shown that the first 3 principal components can account for 90% of the variability. However, the grasp posture data describes the hand only in the final state, it does not describe the trajectory the fingers took to achieve the position around the object. When joint angle data was recorded continuously from subjects performing natural hand movements, 8–9 principal components were needed to describe 90% of the variance (Danziger, 2014). Thus, classifying hand states, and transitioning between fixed postures in a prosthetic is a more straightforward task than attempting the dexterous control of individual fingers (Aggarwal et al., 2011). Although we can treat a robotic effector as part of the body and nervous system of the operator, all current techniques for recording neural activity involve the projection of the high dimensional space of hundreds to thousands of neurons down through the recording electrode array to the much lower dimensionality of the end point of the effector (Carmena, 2013).

### Feedforward control

**Figure 2:**
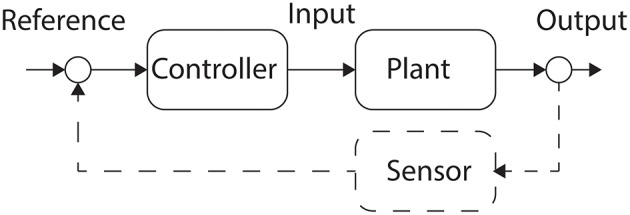
**Feedforward and Feedback Control**. Feedforward or open-loop control is shown here in the solid line. The controller generates a command that is applied to the system, or Plant. In response to the command the system performs an action at the Output. Closed-loop or feedback control is achieved by the inclusion of the Sensor component, shown here as the dashed line. The Sensor measures the Output enabling the Controller to assess the error and adjust the next Input to the Plant.

Feedforward or open-loop control generates a command for the plant that is expected to produce the correct output. However, there is no measurement of the output from the plant, and hence no measurement of error, so the controller has no mechanism to modulate a command (Houk, 1988). A block diagram of open-loop control is shown as Figure 2. Implicit within open-loop control is the assumption of a perfectly described system that can be used to generate a control. Leaving aside the difficulties in creating a perfect model of any system, open-loop approaches do not take noise or measurement error into account.

### Feedback control

Feedback, or closed-loop control requires the inclusion of sensors in the system under control. The feedback controller generates a command for the plant, and the sensors measure the output of the plant in response to the command. If a measurement, such as the angle of a joint differs from the expected output, then the error signal can be used by the feedback controller to modify the generated commands. There are many mathematical approaches that can be used to modify feedback controller output (Crago et al., 1996).

### Adaptive control

Adaptive control can be applied to both feedforward and feedback controllers. By using sensors to measure the input and output of the system adaptive control strategies seek to adjust the controller in response to perturbations in the environment or the controlled system (Crago et al., 1996). Adaptive controllers enable the development of a control strategy without requiring complete knowledge of the system being controlled, however, as a consequence adaptive controllers are rarely optimal.

### Internal model control

**Figure 3:**
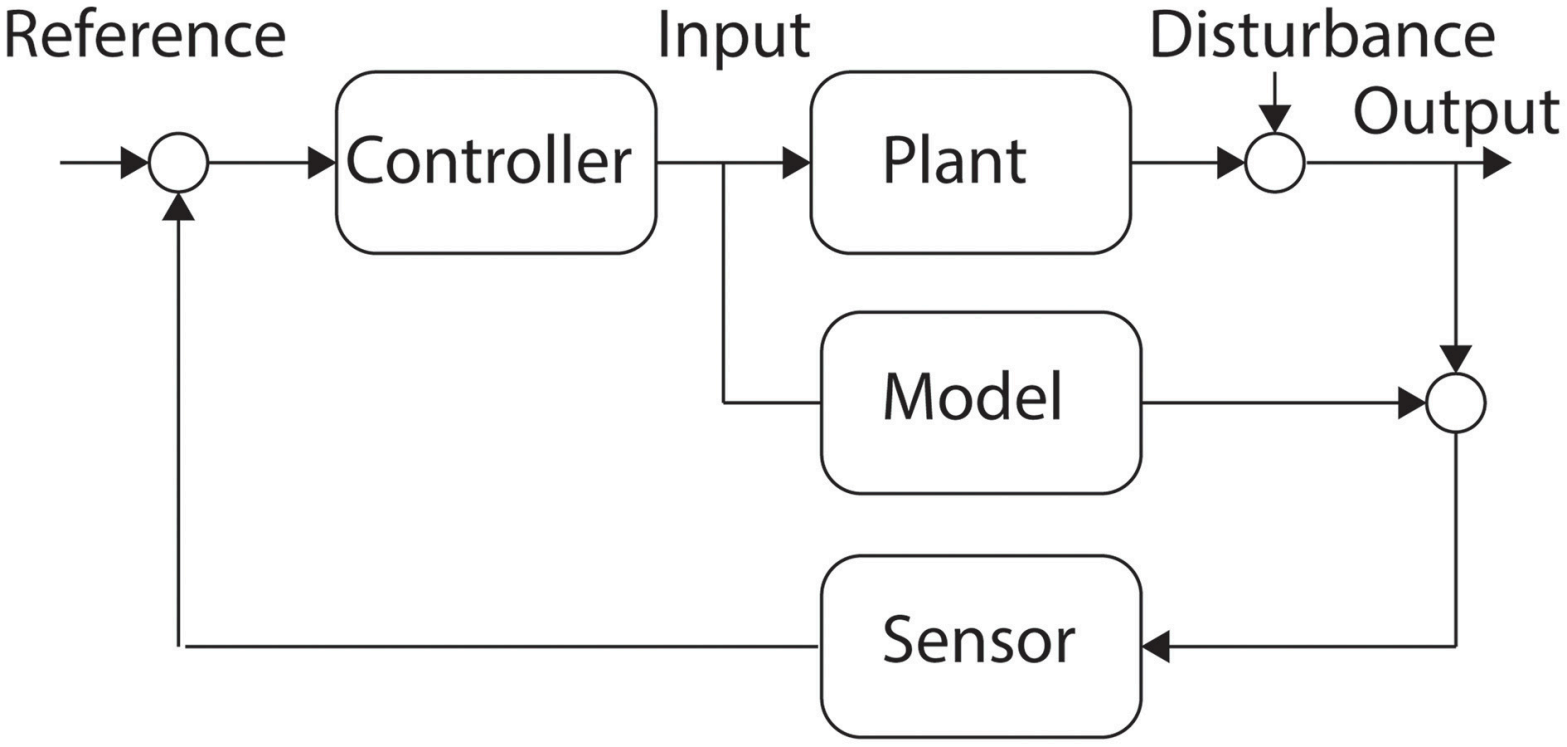
**Internal Model Control**. The inclusion of a model of the Plant allows for the Controller to incorporate some of the dynamics of the system into the control policy.

Internal Model Control (IMC) is an approach to feedback controller design that incorporates a model of the system that is being controlled (García et al., 1989). The model can be developed based only on the relationship between the inputs and outputs of the system, or alternatively a partial model or complete model of the system can be utilized (LeDuc et al., 2011). At each time step the internal model is evaluated forward to a horizon, offering a prediction of the system behavior in response to the controller's input, and the control inputs are evaluated against a cost function to find the optimum command to be executed at the next time step (Pan et al., 2015). A block diagram illustrates IMC as Figure 3.

### Classification

A Classifier breaks a system into discrete states, and maps a relationship between an input and a system state (Schwartz, 2004). Classifiers can be supervised or unsupervised. Supervised systems are trained on an input-output relationship in a data set, and attempt to generalize the relationship to new data, while unsupervised systems attempt to partition or cluster the dataset.

### Actor-critic

The Actor-Critic architecture separates the control policy from the evaluation of the action. The Actor component of the systems choses a policy, which affects the state of the system. The Critic component assesses the state of the system in accordance with a cost function, and provides the evaluation to the Actor (Mahmoudi and Sanchez, 2011). The goal based evaluation differs from the error signal of other control approaches, and does not require that the Actor has a model of the system's behavior (Mahmoudi et al., 2013).

## Control algorithms

Table 1 summarizes the Control Algorithms.

**Table 1:** **Summary of Control Strategies**.

	**Bang-Bang**	**FSM**	**PVA**	**Kalman Filter**	**PPF**	**R-L**	**Feedback Control**	**LDA**	**ANN**	**SVM**	**LSHDI**
FES		Holinski et al., 2013					Ionescu and De Keyser, 2006				
Prosthetic limb		Armiger et al., 2013; Markovic et al., 2014									
DBS	Herron and Chizeck, 2014								Grahn et al., 2014		
Motor BMI			Helms Tillery et al., 2003; Hatsopoulos et al., 2005; Chase et al., 2009	Kim et al., 2008; Li et al., 2009; Dangi et al., 2011, 2014; Gowda et al., 2012; Orsborn et al., 2012, 2014; Corbett et al., 2013, 2014; Jarosiewicz et al., 2013; Marathe and Taylor, 2013; Golub et al., 2014; Matlack et al., 2014; So et al., 2014; Yeom et al., 2014	Shanechi and Carmena, 2013; Shanechi et al., 2014	Mahmoudi et al., 2008, 2013; DiGiovanna et al., 2009; Mahmoudi and Sanchez, 2011	Pan et al., 2015	Zhang et al., 2012; Williams et al., 2013; McCreadie et al., 2014; Iturrate et al., 2015; Xu et al., 2015	Aggarwal et al., 2008; Chang et al., 2009; Shpigelman et al., 2009	Hu et al., 2003; Olson et al., 2005; Olson and Si, 2010	Dethier et al., 2011, 2013
Exo	Cisotto et al., 2013						Nagasako et al., 2015	Xu et al., 2014			
Vision								Peters et al., 2001; Wilder et al., 2009			
Other	Peters et al., 2001; Wilder et al., 2009	Mendez et al., 2013		Marzullo et al., 2010			Shanechi et al., 2013; Iolov et al., 2014	Agashe and Contreras-Vidal, 2011			

Control policies can be implemented by classification, model independent, or model based approaches (Kameneva et al., 2015).

### Control policies with independent models

Consider the system state to be independent at each time step.

#### Bang-bang control

Also referred to as On-Off control, in this scheme when a threshold for a measured variable is crossed a program is activated. Although simple this control scheme has been used successfully to automate tasks that have previously required human intervention, such as the delivery of cortical electrical stimulation after ECog seizure detection (Peters et al., 2001), or the mapping of stimulus thresholds in high electrode count implanted neurostimulators (Wilder et al., 2009).

#### Finite state machine

A State Machine is a model of a system. It can be considered a more complex implementation of Bang-Bang control. The measurement of a system value, combined with the modeled system's current state triggers an action and a state transition (Markovic et al., 2014). If the modeled system is periodic, such as gait during walking, then it can be possible to have transitions due to timing (Holinski et al., 2013), in which case the neuroprosthetic enables state transitions in response to deviants from the periodic behavior, such as starting or stopping the gait.

### Population vector algorithm

The biomechanics of the arm make motor control a difficult problem. Additionally the mechanisim of control within the motor cortex remains an open question. Is the cortical representation in an area such as the activity recorded from M1 encoding the lengthening or shortening of individual muscles (Schwartz, 2004), or is it representing the kinematics of movement (Ajemian et al., 2008)? In either case there is evidence for a forward and inverse model representation of motor control existing within the brain (Andersen et al., 2010; Green and Kalaska, 2011). The Population Vector Algorithm (PVA) is a popular method to decode neural activity recorded from cortical MEA in the motor cortex for the control of a robotic effector, or cursor in a 2D or 3D space. This control algorithm rests on the observation that different neurons have directional preferences—they have higher spike rates for movements in some directions (Shpigelman et al., 2009). Individual neurons do not offer enough specificity to be useful, but a large enough population of neurons, recorded from simultaneously, can be used to determine the intended direction and movement velocity by linear regression. Neuroprosthetic control can then be performed by relying on a “targeting” strategy of decoding the end point trajectory apparent in each cell's activity.

In Helms Tillery et al. (2003) a non-human primate's BMI was extracting an X, Y, and Z signal for the end effector on the robotic arm and the other degrees of freedom of the arm were under the control of the robotic device. In Hatsopoulos et al. (2005) a human participant with 128 electrode array implanted in the precentral gyrus was able to achieve 2D cursor control on a laptop.

Initial implementations of PVA utilized cortical recordings made while the participant watched cursor movement, or the movement of a limb. However, although a PVA decoder created in this way may show good performance in offline testing, the closed-loop performance will not be better, and may be worse (Chase et al., 2009).

### Control policies with dynamic models

There are methods that rely on having a model of the dynamic system describing the parameter the neuroprosthetic is controlling. This might be a model of the spiking behavior of a region of the CNS that is to be modulated by a DBS, or a model of the kinematics of the trajectory of a cursor in a motor BMI, or the kinematics of the gait of an exoskeleton. Models can be linear or non-linear. Using the model, and an error signal, and modeling feedback the next state can be predicted using a variety of methods described below. These approaches are iterative, and work well as part of a closed-loop as they represent the process of the subject modifying the input control signal in response to the feedback signal.

A simple linear state model for velocity can be represented as:

$$\begin{array}{l} x_{t}=Ax_{t-1}+w_{t-1}\\ y_{t}=Cx_{t-1}+q_{t-1} \end{array}$$

where ***x***_*t*_ is the velocity as a 3 dimensional vector at time step ***t***, ***A*** is a matrix of parameters describing the trajectory and ***w***_*t*−1_ is a noise term. The second equation describes the measurement model of the neural activity, ***y***_*t*_. ***C*** is a matrix of parameters that relate velocity to the neural activity and ***q***_*t*_ is a noise term. This model can be expanded with the inclusion of an additional term to model the input of the control signal on the system state as follows:

$$x_{t}=Ax_{t-1}+Bu_{t-1}+\;w_{t-1}$$

where ***u***_*t*_ is the control signal at time step ***t***, and ***B*** is a matrix of parameters describing the trajectory. The task of the following algorithms is to predicte the state in order to generate an error, the difference between the predicted and observed state, which can be used to adjust the control signal.

There are a number of assumptions within the model, including that the sensory feedback to the subject of the current state ***x***_*t*_ is error free and instantaneous (Shanechi and Carmena, 2013). The matrix ***B*** is tightly coupled to a particular task, making it difficult for the subject to use the control input ***u***_*t*_ to drive the neuroprosthetic if the task order changes during use (Matlack et al., 2014). Williams et al. (2013) points to the utility in including both a “hold” state and a “rest” state in the design of tasks for motor BMI decoders, and which is often not included as part of the model state. Finally, Hogri et al. (2015) illustrate a clear box modeling approach (LeDuc et al., 2011) where a simplified cerebellar microcircuit is implemented as a VLSI chip and interfaced to anesthetized rats.

#### Kalman filter

The Kalman Filter (KF) is a recursive optimal estimator and is good at extracting signal from noisy measurements. It has been widely deployed in industrial automation and control systems engineering for over half a century. In its original form the state and measurement models needed to be linear. The extended Kalman Filter (EKF) models non-linear processes where a linearization has been performed, and implementations such as the Unscented Kalman Filter (Li et al., 2009) can utilize non-linear models. A number of variants have been proposed for neuroprosthetic closed-loop control, including SmoothBatch (Orsborn et al., 2012) and ReFIT (Gilja et al., 2012) which capture elements of the neuroprosthetic task in the model and updates the decoder parameters during the operation of the system. Updating the decoder in this manner is referred to as closed-loop decoder adaption (CLDA). In Dangi et al. (2014) Recursive Maximum Likelihood is used as part of CLDA to continuously adjust the KF. This is probably useful because the recording of the neural signal may be non-stationary due to factors (electrode drift, movement artifacts, external noise) as well as the fact that the subject may have changes in attention during the operation of the device, and the learning process may change the parameters for error and modeling (Chase et al., 2009). When examining various parameters that can be tuned in the decoder, (Cunningham et al., 2011) determined that bin width has a large impact on the performance of the KF, and should be optimized. Potentially due to the subjects ability to interact with the closed-loop system, shorter bin widths of 25–50 ms provide improved performance over longer bin widths.

#### Point process filters

The activity of individual neurons in the ensemble can be modeled as point processes with each spike being an event, which enables the filter to respond much more rapidly then methods that rely on binned spike counts or estimates of instantaneous firing rates (Li et al., 2009).

### Reinforcement-learning

In Actor-Critic architectures two coupled systems work together with complimentary models of the task. The two systems adapt using a Reinforcement Learning approach (DiGiovanna et al., 2009). The user of the system supplies a signal indicating success or failure to the Critic, which supplies a training signal to the Actor to allow adaption (Mahmoudi et al., 2008). By trial and error the Actor interacts with the environment, and the Critic's feedback rewards successful actions. The Actor-Critic approach may also be well-suited to neuroprosthetic control in a real world usage scenario where the task and associated trajectory varies from moment to moment, and achieving the goal may be the only reinforcement signal available (DiGiovanna et al., 2009). Mahmoudi et al. (2008) describes a neuroprosthetic for a Sprague Dawley rat with 32 electrodes implanted in primary motor cortex (M1)—symmetrically, 16 electrodes per hemisphere. This was used to control a robotic arm, which the rat used to press levers. Meanwhile the Critic component is implemented as a computer agent that adapts via the Reinforcement Learning paradigm based on the rewards the rat user receives, and the rat user is learning to modulate its neural activation modifying the directional tuning of the units in M1. An extension to this approach involves extracting the goal success signal directly from the subject. By recording from the Nucleus Accumbens in the ventral striatum of rats, an area believed to associate sensory perception with motor tasks, (Mahmoudi and Sanchez, 2011) were able to use the rat subject's internal representation of goal success as the evaluative feedback signal to the Critic component.

#### PID feedback control

The Proportional-Integral-Derivative (PID) controller is an extremely common and widely deployed controller in closed-loop systems. The three terms—proportional, integral, and derivative are calculated from the plant's response to the input and are summed to generate the error signal (Åström and Wittenmark, 1990). Chaos control approaches, such as delayed feedback control, utilize the dynamics of the system to modify the control input. By taking advantage of the chaotic system sensitivity to perturbation, system state can be changed with minimal cost. In Vlachos et al. (2016) the use of delayed feedback control enables closed loop control of a seizure model (a spiking neural network) and the recovery of the non-seizure dynamics, while in Slutzky et al. (2003) seizure activity induced in rat hippocampal slice preparations was moderately controlled.

### Control policies with classifiers

Classifiers don't need a model of the system instead they attempt to determine a relationship between a set of measurements and a given state. Some classifiers can be sensitive to changes in the data they use to determine the classes (Lotte et al., 2007), such as Artificial Neural Networks whereas Linear Discriminant Analysis is more robust in the face of changes to data used to train the classifier.

#### Linear discriminant analysis

Linear discriminant analysis (LDA), and other related techniques, are statistical methods that find the features in the measurement of a signal that indicate the probability that it belongs to a given class (Mika et al., 1999). The assignment to a class can be used to trigger a neuroprosthetic intervention, such as the detection via EEG of a motor command and the subsequent activation of an ankle exoskeleton (Xu et al., 2014).

#### Artificial neural network

**Figure 4:**
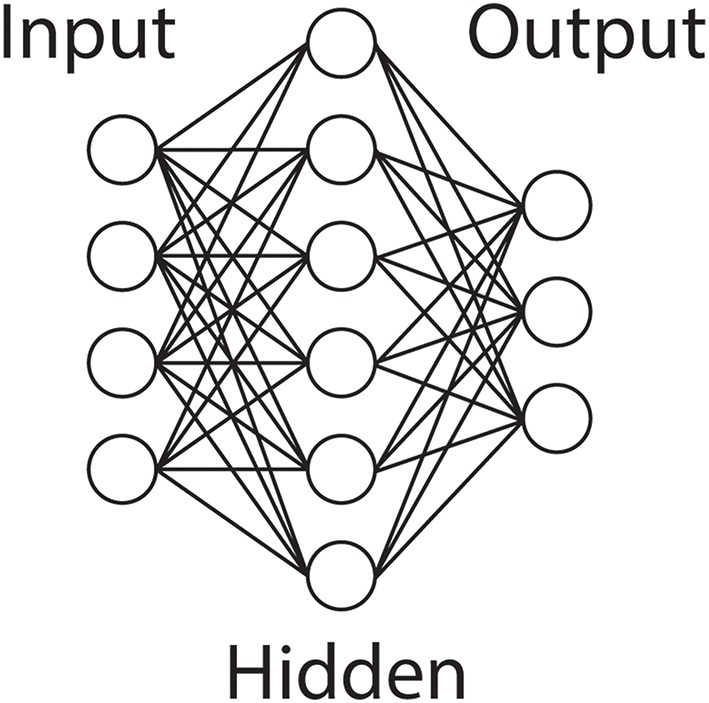
**Artificial Neural Network**. An illustration of a typical ANN topology. An input layer projects to a single hidden layer, which connects to the output layer. Common variations include additional hidden layers and recurrent connections.

The Artificial Neural Network is a data driven approach to classification that in contrast to LDA and other statistical methods does not rely on the assumption of the underlying probability distribution of the system (Zhang, 2000). ANNs are organized in layers, with nodes or neurons connected typically in an input, hidden and output layer structure (Figure 4). There are numerous topologies, but among the most popular is the Multi Layer Perceptron (MLP), a three layer feedforward network. ANNs are trained with the presentation of input data that has been identified as belonging to an output class, and a learning rule is applied to adjust the weights on the connections between the nodes, of which back propagation is the most well-known.

ANNs have been used to predict end-point gait parameters from the EMG recorded from the neuromuscular activation of subjects with Spina Bifida (SB; Chang et al., 2009), and to achieve realtime dexterous control of a myoelectric prosthetic hand from cortical recordings of rhesus monkeys (Aggarwal et al., 2008). Echostate Neural Networks are a Recurrent Neural Network (Sussillo et al., 2012) that have been used in non-human primates for a motor BMI, and have been able to outperform the Kalman Filter.

#### Support vector machines

The Support Vector Machine (SVM) is a supervised machine learning approach that can perform classification or regression. The SVM identifies a hyperplane that separates classes within the data, by non-linearly projecting data points into a higher dimensional space (Tapson et al., 2013).

#### Linear solutions to higher dimensional interlayers

Linear Solutions of Higher Dimensional Interlayers (LSHDI) are a class of networks that have some similarity in architecture to ANNs, with an input, hidden, and output layer. They are distinct due to the much larger hidden layer, the random generation of the weights on between the input and the hidden layer, and the linear response of the output layer (Tapson et al., 2013). The Neural Engineering Framework (NEF) builds systems out of networks that have LSHDI characteristics (Eliasmith and Anderson, 2004). The NEF has been used to design Spiking Neural Networks that implements the Kalman Filter as part of a cortical motor BMI (Dethier et al., 2011). The Synaptic Kernel Inverse Method (SKIM) is an LSHDI network for spiking input (Tapson et al., 2013) that can perform both classification and regression.

## Feedback

There can be more than one feedback loop in the neuroprosthetic system (Broccard et al., 2014). Feedback can be the visual observation of the robotic effector as it is in many motor BMIs, allowing the operator to modulate their neural activity before it is decoded. Or in the case of a DBS neuroprosthetic the feedback signal may be acquired from recording electrodes implanted alongside the stimulating electrodes, in which case the feedback signal is returned directly to the device (Herron and Chizeck, 2014). Bidirectional interfaces, via the PNS or the CNS, enable the transformed signals of sensors on the robotic effector to be transmitted into the operators nervous system and interpreted as a sensory percept (Armiger et al., 2013). Feedback can also be from sensors on the robotic effector directly to the controller, bypassing the operator (Broccard et al., 2014; Markovic et al., 2014). And finally, reversing the direction of information, natural sensors can be used as a source of feedback for the controller (Holinski et al., 2013; Mendez et al., 2013; Wright et al., 2015) and the brain can be used as the source of the error signal (Mahmoudi and Sanchez, 2011).

## Discussion

We have reviewed the control policies employed by recent neuroprosthetic systems. For the purposes of this review we included motor BMIs, assistive devices, neuromodulatory systems, and other devices with an interface into a subject's nervous system. Many of the devices reported on in the literature are being developed in non-human primate or other animal models, only a subset have been tested in human subjects. There is a frequent conflation of detection and classification approaches with digital or Bang-Bang control within the literature.

Although closed-loop systems have been demonstrated experimentally there remain significant limits on our ability to describe the activity in the brain, and consequently develop control policies to respond to that activity. Simulation of cortical activity (Ehrens et al., 2015; Sandler et al., 2015; Vlachos et al., 2016), the use of experimental platforms (Keren and Marom, 2014), and the use of animal models has enabled the development of a wide range of neuroprosthetic systems. However, the appropriate method to transition these systems in human subjects is not clear. Among the difficulties is the body's response to chronic implantation of microelectrodes (Fernandez et al., 2014), the appropriate place to collect a signal (Krook-Magnuson et al., 2015), and the possibility that longterm attempts to control a cortical system may compromise some of the desirable behavior (Keren and Marom, 2014).

Comparisons of control policies across different modalities, interfaces and levels of invasiveness are difficult. Even within a given neuroprosthetic category it can be difficult to perform a comparison due to the wide variety of task designs, different subject training regimes and varying reported metrics; (Koyama et al., 2010; Sussillo et al., 2012) are rare exceptions. Examination of Table 1 reveals that the motor BMI discipline appears to have explored the widest variety of control policies, and that the use of the Kalman Filter as part of a closed-loop system has broad support. Improvements to the traditional Kalman Filter to allow non-linear models of neural activity combined with its ability to be implemented in real-time continue to make it an attractive approach.

Closed-loop motor BMI systems have had significant success with closed-loop decoder adaption (CLDA; Shanechi and Carmena, 2013), supporting the use of closed-loop control. However, the CLDA approach has identified two distinct strategies in motor neuroprosthetics—decoding vs. learning. The decoding approach aims to read the natural motor plan whereas the learning approach monitors the changing neural activity as the brain learns to operate the prosthetic. It is not known at this stage if a similar duality of strategies will be applicable in other modalities. The difficulty in specifying a model for use in many of the control policies previously describes arises from our continued uncertainty about specific action of many of the components of the nervous system. It has been observed that users of Cochlear Implants have improved speech recognition performance after completing training with the device (Doucet et al., 2006), which may argue for a learning interpretation. An important caveat for the learning approach is that the neuroprosthetic system must be stable as regards the interface and transform of the input signal to the effector output, to allow the subject the opportunity to develop the “prosthetic motor memory” necessary for skillful operation (Carmena, 2013).

Neuroprosthetic development of closed-loop systems has been driven in part by the concern that the risks involved in highly invasive interfaces need to be mitigated by a strong case for the therapeutic benefit. Patient abandonment for upper limb prosthetics is high, with many wearers ceasing upper limb prosthetic use within 12 months of receipt of the device. Concerns cited by users are weight, appearance and difficulty of use (Biddiss and Chau, 2007). Extrapolating to more invasive systems, it may be difficult to argue the cost benefit if patient dissatisfaction is very high. Although these devices cannot be abandoned in the same manner as a detachable prosthetic limb, there is some suggestive research indicating an unwillingness to participate in experimental trials, which may suggest that the perceived benefit of neuroprosthetic systems by the target patient populations remains low (Illes et al., 2011). By improving control we can offer improved functionality and increased therapeutic benefit.

## Author contributions

JW performed the review and drafted the manuscript. JT, AS, and VM revised the manuscript.

### Conflict of interest statement

The authors declare that the research was conducted in the absence of any commercial or financial relationships that could be construed as a potential conflict of interest.

## Appendix: literature search methodology

To generate a list of papers for the review we performed a search on Scopus using the following search criteria, restricting the results to articles or conference proceedings:

Topic = (neuroprosthetic OR neurorobotic OR exoskeleton OR neurostimulator OR (brain AND interface)) AND Topic = (control OR controller AND (scheme OR algorithm OR strategy)) AND Topic = (closed-loop OR closedloop). The results were limited to Articles and Conference Proceedings from 1990 to 2015.

With the above search we obtained 147 papers from Scopus. We excluded papers that were from unrelated fields, as well as papers that described the design or fabrication of an exoskeleton without reference to a control system, or that described a control strategy used by an exoskeleton or rehabilitative device without a nervous system interface. We excluded papers that discussed computing architectures for experimental design, or detailed the fabrication of electronics. Once duplicates and excluded papers were removed we were left with 85 articles.

The papers were divided into five categories based on their primary topics.

### Control strategy

Papers mainly describing the design of an algorithm. Papers comparing the performance of algorithms by a particular neuroprosthetic. Papers describing a change to the control loop such as the addition of feedback or the use of additional signal processing.

### Neuroprosthetic design

Papers describing a neuroprosthetic system, including interface site, electrode fabrication, sensors, architecture and algorithms.

### Device testing

Papers that describe the performance of an experimental neuroprosthetic. Papers that describe an experimental neuroprosthetic in a non-human test subject. Papers that compare the performance of a neuroprosthetic in a patient with an existing clinical therapy.

### Simulators

Papers that describe a simulation environment for development or testing of neuroprosthetics, or papers that describe a model of the nervous system for a neuroprosthetic device or control algorithm to interact with.

### Reviews

Papers that are reviews of neuroprostheses, algorithms or interfaces.
